# Developing and implementing a geriatric surgery co-management program for older adults: insights from document analysis

**DOI:** 10.4081/sigaf.2025.28

**Published:** 2025-10-20

**Authors:** Abigail Baim-Lance, Fred Ko, William Hung, Minji Kim, Kavya Sreevalsan, Stephanie Chow

**Affiliations:** 1 Icahn School of Medicine at Mount Sinai, New York (NY); 2 James J. Peters Department of Veterans Affairs Medical Center, Bronx (NY); 3 University of Minnesota School of Medicine, Minneapolis (MN); 4 Ohio State University Wexner Medical Center, Columbus (OH), USA

**Keywords:** geriatric surgery co-management, document analysis, implementation strategies, program development

## Abstract

The *Aging, Life Innovations, Goals and Needs-Collaboration Achieving Readiness and Empowerment* (ALIGN-CARE) ambulatory geriatric surgery co-management is an interdisciplinary program to better prepare frail older patients for surgery. We examined the development and implementation of this complex clinical program using the Medical Research Council (MRC) evaluative framework, document review, and analysis of program meetings. We analyzed 84 meetings (81.5 hours) generated at four recurring meeting types (advisory, interdisciplinary, surgeon, and research) between January 2020 and January 2022. Documents were coded and thematically analyzed to develop the implementation timeline, evolving program components, and implementation processes supporting program development. ALIGN-CARE evolved and stabilized four distinct components (referral, scheduling, assessment, and plan), drawing upon several implementation strategies. Each meeting drove the process in unique ways. Program flexibility and responsiveness were key ingredients driving implementation. ALIGN-CARE has matured through the adaptation of program components, with stakeholder meetings supporting these objectives. The document analysis method identified processes key to developing geriatric surgical co-management and advancing complex interdisciplinary programs for older adults.

## Introduction

Major surgery is a common intervention in adults over 65 years, but risks increase with age, related to loss of function and independence due to the adverse consequences of surgery.^1^ Older adults with complex chronic conditions and frailty^2–5^ and mixed functional status^6^ are more likely to have poorer surgical outcomes. Surgery and subsequent care require taking into account these complexities and the potential hazards^7^ introduced by surgery, hospitalization, and care transitions. Geriatric co-management, defined as collaborations between geriatric and non-geriatric teams, can assess and manage vulnerabilities, including chronic co-existing diseases, polypharmacy, functional dependency, and malnutrition. In the surgical context, geriatric co-management can reduce poor outcomes throughout the pre-, peri-, and post-surgery continuum.^8–10^

Establishing and implementing a geriatric-surgery co-management program is complex, involves multiple interacting components over time,^11^ and requires: i) collaboration across geriatricians, social workers, administrators, surgeons, primary care providers, patients, and caregivers; ii) delivery of timely and multiple interlocking interventions; and iii) incorporating a high degree of flexibility across a care continuum to support planning for older patients’ diverse clinical and social needs. The American College of Surgeons (ACS), recognizing and prioritizing the unique needs of the geriatric population during surgery, has standardized the care approach with the ACS Geriatric Surgery Verification (GSV) program. This program comprises a set of 32 comprehensive standards that must be met to receive a geriatric verification. Limited uptake of the ACS GSV program has been observed^12^ due to intervention and implementation complexity.

The *Aging, Life Innovations, Goals, and Needs-Collaboration Achieving Readiness and Empowerment* (ALIGN-CARE) ambulatory program is a pragmatic intervention that optimizes care for older surgical patients by integrating geriatric principles into existing surgical pathways to enhance surgical experiences and outcomes. ALIGN-CARE was developed within a large tertiary hospital setting in New York City. ALIGN-CARE assessed its first patient as part of a trial in January 2020, and over the next two years, the program invested significant start-up efforts in developing program components and activities to effectively integrate geriatrics and presurgical processes. Currently, ALIGN-CARE consists of an interdisciplinary geriatrics team (geriatrician, nurse practitioner, social worker, scheduling administrator/care coordinator, and geriatrics trainees) focused on four program components (surgeon referral, patient scheduling, pre-operative patient assessment, and care planning) with the aim of providing interventions targeting and mitigating pre-operative risks to optimize peri-operative outcomes for older adults (Supplementary Table 1).

While geriatric surgery co-management has been associated with positive outcomes, such as reduced hospital length of stay, postoperative complications, and mortality, program implementation is largely confined to hospital settings.^13–22^ Moreover, programs are rarely evaluated through the lens of their development and implementation, with only one known exception in the geriatric-surgery space (SURGE-Ahead to create a digital application with AI-enhanced suggestions to display evidence-based recommendations for geriatric co-management).^23^ Our qualitative analysis responds to this gap by examining ALIGN-CARE development and strategies to implement the intervention within a complex health system environment amidst real-world challenges. We draw upon the Medical Research Council (MRC) framework^11^ for evaluating complex interventions to understand two areas of program development: i) intervention components defined as the “what” of delivering complex geriatric-surgery co-management (*i.e.*, intervention elements, activities, and tasks); and ii) implementation processes or “how” to develop the program effectively (*i.e.*, meetings and other strategies to support program implementation). We leverage document review and analysis^24^ of program meetings as “naturally occurring” program data to understand its development, including non-linear and emergent aspects. Clarifying program components and processes of ALIGN-CARE integration into a health system environment will illuminate how to develop and deliver these and similar complex interventions on behalf of the care of older adults across health systems.

## Materials and Methods

### Data sources

We reviewed 84 meeting minutes (81.5 hours) generated at four types of recurring program meetings between January 2020 and January 2022 to assess program development (Table 1). Minutes were not produced as an analytic product, but as a program tool to keep track of discussions and action steps. ALIGN-CARE held four regular meetings with the following groups: 1) interdisciplinary team (IDT); 2) department advisors; 3) surgery teams; and 4) researchers. Each meeting used an agenda developed by the Program Director (PD), the only regular attendee of all meetings. The agenda was a template to populate a set of key meeting points, including items achieved, challenges, and strategies to mitigate. IDT and advisory meetings covered ALIGN-CARE and related geriatrics services for older adults at elevated risk for health complications, hospitalizations, and premature mortality. The PD took meeting notes at advisory, surgery, and research meetings, and the social worker took notes at IDT meetings. They uploaded notes to a shared drive.

### Documentation analysis procedures

A team trained in qualitative analysis participated in meeting minutes review and coding following the document review and analysis procedures. Document review is a qualitative approach that utilizes naturalistic materials to gather information about decision-making processes, activities, and the individuals involved, as well as the contexts in which they occur.^24^ Documents are treated as qualitative text in a fashion similar to the treatment of interview or focus group data. In this case, the team initially established a coding schema consisting of five domains of interest consistent with the MRC complex intervention framework: meeting purpose, program components, adaptations, management activities, and barriers and perceived value/facilitators of the program as a whole and by component. In two iterations, team members independently reviewed and coded the same meeting record using the coding schema, extracting from meeting minutes specific content that corresponded with the code definitions. Our team compared extractions and collectively reached an agreement on code meanings, adding codes as necessary. Once consensus was achieved, we proceeded to assign meeting notes by type to each coder who extracted meeting data into a spreadsheet. Each coder then summarized their findings in a pattern-seeking memo. Memos were compared and discussed to identify and visualize themes across meetings related to ALIGN-CARE development, barriers and facilitators, and implementation strategies used to overcome challenges, as well as how each meeting type supported program development. This analysis is part of STUDY-21–01766, approved by the Mount Sinai Institutional Review Board.

## Results

### ALIGN-CARE program development

#### Timeline

**Figure 1.**
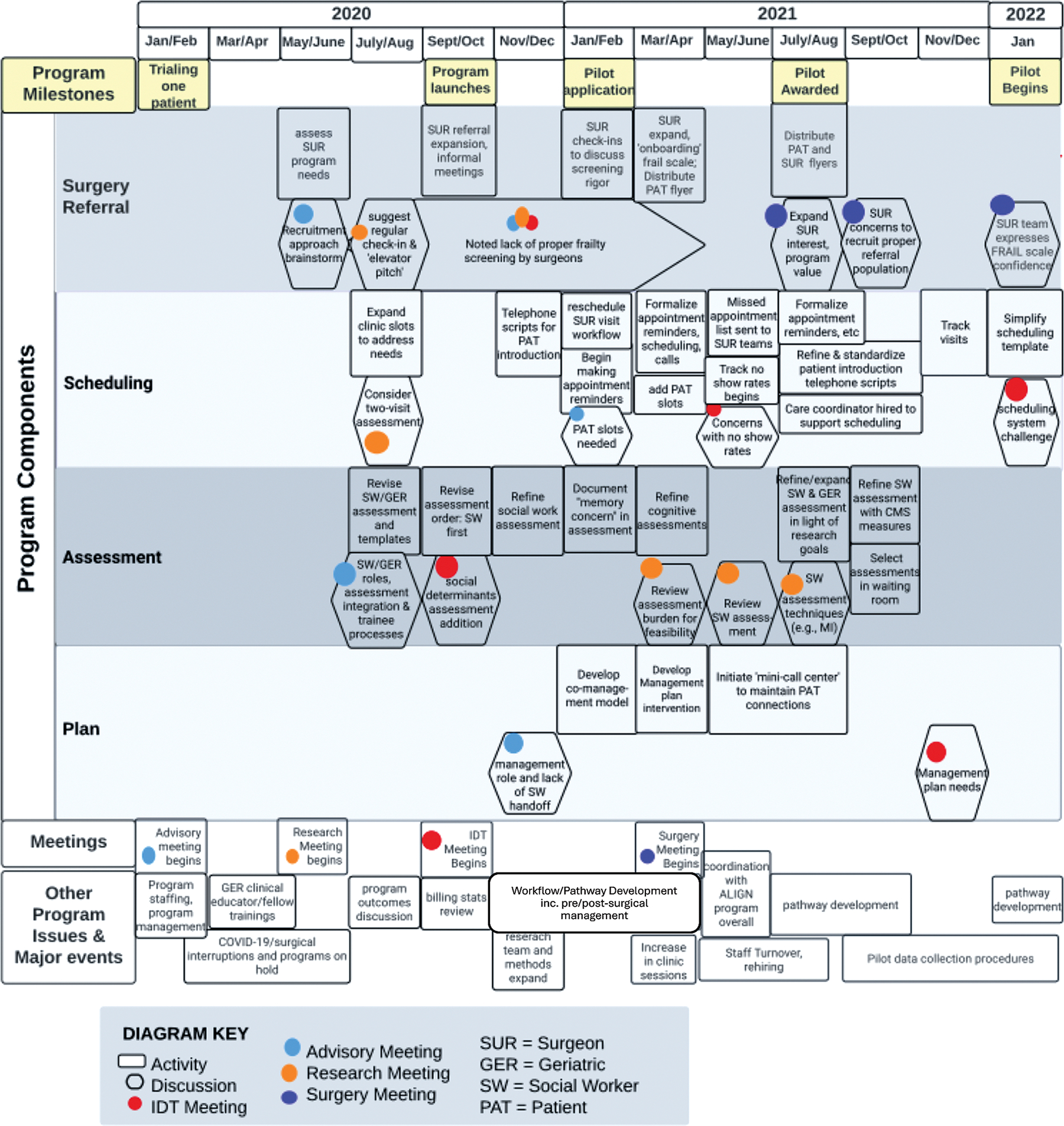
ALIGN-CARE program components and development timeline.

The ALIGN-CARE timeline (Figure 1) shows how each of the four program components developed between January 2020 and January 2022 (2020=Year 1, 2021=Year 2). ALIGN-CARE, as a surgical co-management model, began planning in January 2020, started regularly assessing patients in September, and refined the model over the following year to solidify its components. Each phase included activities, from additional program delivery details to implementation strategies, aimed at increasing program adoption and responding to emerging challenges and opportunities. In Year 1 (2020), the program focused on surgeon referral, patient scheduling, and developing the comprehensive geriatric assessment. The first year was also marked by the COVID-19 pandemic, initially a time with enormous competing demands in the health system, which may explain limited activities that rapidly accelerated in the second half of Year 1 and into Year 2 (2021). A key focus in Year 2 was on developing the care management approach, including planning recommendations based on the geriatric assessment and strategies for supporting patients without directly taking over their care management.

Our data show program meetings as active spaces to surface and tackle different issues. Overall, meeting discussions led to resolution within 1–2 months; yet, several recurring problems were not completely resolved (surgeon referrals, patients not showing up to visits), suggesting ongoing efforts are needed. A major milestone was achieved with the receipt of a pilot study award in July 2021, building upon the prior and ongoing efforts of the research team to standardize program components and streamline data extraction processes aimed at supporting program improvement and organizational learning.

The following section describes program components and emergent activities as they pertain to each ALIGN-CARE component: surgeon referral, scheduling, assessment, and plans. We examine how the meeting, as a specific implementation strategy, supported responsive program development (see Figure 1 for details on program component activities and implementation strategies).

#### Program component development

1. *Surgery referral*. The first step of ALIGN-CARE is the surgeon team conducting a frailty screening and then referring eligible patients to the ALIGN-CARE geriatric team. From early on, worries of “improper screening” and a lack of referrals were raised in IDT, advisory, and research meetings. IDT team members suggested including a greater number of referring surgeons in ALIGN-CARE (September 2020, March 2021), and advisors suggested setting up a regular “check-in” with the surgical team and developing a clear “elevator pitch” of the program’s value and purpose, which resulted in informal meetings between the geriatrics team and surgeons and a formal monthly meeting starting in April 2021. In surgery team meetings (total 5), participants discussed referral challenges on the patient side, stating there were “difficulties to get proper populations for referral” and the “extra ALIGN-CARE visit increases burden on patients who often have to travel a long way” (February 2021). These notes also glean the differential uptake across surgical sub-specialties, with one team making referrals easily while another had “barely enough time to open a note to start the referral” and found the system “cumbersome” (March 2021). The geriatric team responded in several ways, including conducting surgeon team training and producing surgeon-facing flyers and patient-facing brochures with information about the program. Despite these efforts, concerns continued about surgical engagement. Advisory meeting attendees in April 2021 suggested finding other places to post recruitment flyers. Meanwhile, in ongoing research meetings, discussions have continued around the perceived “accuracy” of frailty scale usage by busy surgeons, as well as how best to “pitch” the program to gain their support (October 2021). Some of these efforts may have paid off; surgeons seem to express more confidence in using the frail assessment in the January 2022 meeting.
2. *Scheduling visits*. Scheduling involves following up on the surgical team’s referral to arrange the assessment with the geriatrics team. Several months after initiating the program, active conversations arose about scheduling needs and challenges, including patients not showing up for their appointments. Other issues affecting scheduling were staff turnover, insurance, billing logistics, and finding clinical space and examination rooms to conduct assessments (June 2021). Scheduling issues prompted the highest number of improvement activities across the ALIGN-CARE components, with most occurring in 2021. Activities included: appointment reminders (January 2021); a quality improvement project to reduce no-show rates by improving the workflow (June 2021); reviewing geriatric appointment slots going unfilled (April 2021, 2 meetings); standardizing the telephone script for outreach (October 2021); dedicating staffing to focus on scheduling (July 2021); and refining a scheduling tool (January 2022). The team discussed the benefits of scheduling software (January 2022). Advisors encouraged the program to track appointment attendance rates (January 2021), which began in May 2021.
3. *Assessments*. Once appointments were scheduled and attended, the geriatrics team conducted a comprehensive assessment incorporating geriatric and social work domains. Assessment type and quality developed throughout the period. An important discussion was the unique inclusion of the social worker’s determinants of health assessments (October 2020). This discussion focused on identifying templates and tools, avoiding screening redundancies, and streamlining workflows, including the advantages of a second visit to split up the assessment workload. The research team contributed to identifying the optimal assessment format, exploring (though ultimately not adopting) a web-based tool (July 2021). The research team overall characterized the intervention as “assessment heavy”, which they thought was helpful but possibly a challenge clinically; the team recommended finding a feasible and sufficient “sweet spot”. The advisory also focused on standardization, integration, and “compromise” between clinical and research needs beginning in August 2020. It led the program a year later to “revise templates to reflect compromise between clinical and research instruments” (August 2021).
  Research meetings also emphasized the importance of data collection, with frequent references to REDCap software for building a clinical and research-based database. The REDCap implementation went from being “considered” (August 2020) to “decided on” (June 2021) over a 9-month period. An implementation science focus emerged in December 2020 during research meetings, marked by the addition of an implementation scientist to the team who brought an emphasis on learning to inform program scaling.
4. *Management plans*. The geriatric and social work assessments result in a management plan for the patient, the surgeon, and potentially the primary care physician or other community-based activities. While the focus was consistently on conducting comprehensive geriatric assessments in preparation for surgery, by late 2020, advisors asked how ALIGN-CARE might supplement primary care by providing recommendations, particularly in cases where no existing social worker was available. Establishing program parameters was stressed but also somewhat fraught; the notes state that, “If we are not taking on the medical care, then social work shouldn’t take on care either” (November 2020). Six months later, in June 2021, advisors further emphasized the importance of a “care coordination and care plan” to not “lose what has been achieved in the program” and suggested a “mini-call center” (June 2021) as a midpoint between consultation and co-management, which was implemented as a phone triage hotline.

### Issues that cross-cut program components

#### Consolidation into a pathway

In addition to specific component development, meeting discussions included higher-level program frameworks. IDT discussed the overall ALIGN-CARE workflow in November 2020, followed by continuous review of modified workflows and frailty pathways on a near-monthly basis in early 2021 (October-December 2020, March 2021). Research began supporting a visual pathway mapping exercise by mid-2021, which currently guides the program.

#### Program/role definition

Advisory meetings provided guidance on defining ALIGN-CARE responsibilities as distinct from other geriatric clinical programs, as well as the roles of the interdisciplinary team members (November 2020). They also focused on the role of the surgeons (January 2021), which was echoed in the research meeting discussions. In surgery meetings, there was continued discussion about the role of the surgical team and ways to help them feel more involved in program investment. Research meetings included discussions on which program roles could be leveraged to incorporate data collection tasks (September 2021).

#### Training and education

An emphasis was placed on identifying barriers to ALIGN-CARE implementation, and training and education were common mitigation strategies. IDT focused on training administrative staff on clear, standardized patient messaging, geriatric medicine fellow trainee didactics, education on high-risk geriatrics care, interdisciplinary teamwork, and quality improvement (October 2021). Advisory and surgeon meetings discussed surgeon onboarding *via* informational pamphlets and flyers, both surgeon- and patient-facing, and ongoing feedback. Research team meetings focused on the importance of training in standardized data entry to assess program fidelity.

## Discussion

While ALIGN-CARE originated with a strong rationale and basic components, its highly iterative two-year start-up demonstrates that there is a need to work out additional specific elements, as well as “behind-the-scenes” implementation strategies to drive the program forward. We separate the discussion into the program’s “what” (program elements) and “how” (implementation strategies), but it is important to recognize that they worked in concert in support of overall program evolution.

### The “what” of a complex geriatric-surgery co-management program

Our analysis has yielded several insights into the implementation of an outpatient geriatric-surgical co-management program, building upon and extending the results we previously presented (Table 2).

At the point of referral, the initial element of surgeon-delivered frailty screening did not change. However, involving non-geriatric specialists in geriatrics-led initiatives also required direct education and communication with patients. Without face-to-face opportunities, flyers or other patient-facing informational materials are important, as other studies have also demonstrated.^25^ These materials may also indirectly increase providers’ awareness of the program – especially when used alongside provider-focused implementation strategies (see next section).

Scheduling has proven to necessitate the most precise and coordinated efforts to guarantee that patients are appropriately scheduled and attend the geriatric assessment. Although coordination aspects are often perceived as simple, existing literature emphasizes the importance of robust linkages^26^ and seamless transitions.^27^ The program learned this and put into place several actions for scheduling, including recruitment scripts and appointment reminders, activities recommended in the literature to optimize delivery efficiencies.^28^

Assessments and management plans underwent ongoing refinements. Attention to the specifics of the plan was identified as a necessary component after performing geriatric assessments; furthermore, supporting patients to implement their plans requires additional effort. Troubleshooting weak linkages between primary care and surgical teams to hand off crucial pre-surgical information frequently occurred, which is consistent with interprofessional alignment barriers identified in the literature regarding the effective implementation of geriatric assessments^29^ and plans.^30^ The decision to establish an ALIGN-CARE hotline aligns with some innovative models,^31^ but the specific application is novel and promising in finding a middle ground of remaining involved in patient care without assuming full-time management.

### The “how” of a geriatric co-management program: strategies to support implementation

The arising challenges required additional “behind the scenes” implementation strategies, alongside direct program delivery adaptations (Table 2), which we can map to the Expert Recommended for Implementing Change (ERIC) strategies,^32^ a set of 73 implementation strategies to promote program intervention success. While ERIC was not explicitly used to select strategies, the team naturally implemented several of them, including the development of provider-facing educational materials, the collection and review of quality data, the establishment of learning meetings, and the engagement of outside “data” experts.

In this process, establishing learning meetings – itself an implementation strategy – formed a meaningful basis to generate the additional strategies. Each of the four meetings exhibited a unique emphasis and contribution, from surfacing workflow and logistical issues (IDT, surgeon) to higher-level strategizing (advisory), to completing the learning cycle by making items explicit and measurable through the involvement of researchers. Holding separate, concurrent, and staggered meetings over time meant that ideas emerging in one meeting were advanced in another; for example, “standardizing elements and pathways” arose from the research meetings, which subsequently featured in IDT meeting discussions to address practical implementation issues. Programmatic changes (or barriers to making them) were then reported out and revised with input during advisory meetings. Emphasis on “compromise” – a recurring sentiment between advisors and researchers – occurred by having diverse experts but finding common ground through the overlapping attendance of the PD, who translated issues and supported moving the program forward.

Surgery meetings, themselves arising as a specific strategy, brought to light barriers to implementation and boosted communication to improve buy-in and lead to providing additional education materials. Attending meetings also deepened understandings of one another and how to overcome challenges. Some meetings – with surgeons and the IDT – enhanced program buy-in. The meetings provided opportunities to reinforce shared commitments and goals. In doing so, ALIGN-CARE emulated greater collaborative practice, another strategy found to foster successful team practice.^33^ The infrequent and uneven nature of the surgery meetings – with many involving research but few including surgeons and inconsistent participation from surgical leaders who might have lent greater weight to the initiative – underscores the difficulty of securing meaningful engagement in the face of competing demands and time pressures. This could also be a good chance to consider, with more careful thought about the benefits of these meetings, putting in more focused effort or holding flexible meetings.

### Document analysis as a learning source

Document analysis helped our team identify program development – the “what” and “how” – as it unfolded over time through a dynamic and iterative process. Real-time, naturally occurring data meant that collection imposed little burden on busy clinical staff and captured immediate activity instead of delayed reports. In keeping with the MRC framework, we were also able to observe both linear and non-linear processes, as well as episodes of team creativity to make progress within complex and routinized health systems.^34^ We identified time lags and periods of heightened activity, as well as persistent challenges, unresolved issues, and instances of overlapping or disconnected discussions. We also noted the use of multiple meetings as a deliberate implementation strategy, observing how members, tasks, and conversations contributed to adaptive responses.

Given the successful insights gathered about ALIGN-CARE development, this method begs several application-oriented considerations for use in general health program development. The first is whether a closer connection might be forged with the principles of continuous learning process and quality improvement.^32^ Document review, as a qualitative data source, may be beneficially added to the qualitative toolkit.^35^ Second, to what extent can records, if collected with the idea of analyzing them, be documented more rigorously or in more standardized ways towards real-time evaluation and intervention at an earlier point? Third, in this development process, the two-year span suggests that a relatively long runway was needed to make responsive adaptations.

Building on our findings, we should also examine the timeline and nature of the iterations undertaken by a geriatric-surgery co-management program that begins with the adapted ALIGN-CARE model and incorporates implementation guidance.

### Limitations

Documents offer a window into program development, but they were not written with the intent of being used for analysis. While “naturally rich,” these notes may also be limited by the fact that they are often written quickly, with a primary focus on decision points and next steps. It is sometimes difficult to determine whether a problem that led to a proposed mitigation strategy actually materialized – especially if subsequent notes do not indicate a resolution or clarify the time elapsed between the issue and its solution. These limitations make achieving complete accuracy challenging. Looking ahead, we might improve the depth and reliability of documentation by using recordings, capturing more detailed observations, or adopting a more structured note-taking approach. A second limitation was the inability to capture all the “actual work” of the program if it fell outside of the meeting notes. Multiple meeting documents allude to informal meetings without written records. Thus, what we have offered may not be a total picture of the process or outcome. However, we can never attain a complete picture in any research, and there are always blind spots in a dataset. Further, notes clearly captured challenges and responses in some detail, which we have then used to characterize a dynamic and adaptive process with specific outcomes.

## Conclusions

Through document analysis, we identified key evolving elements in the geriatric-surgery co-management model, as well as implementation strategies to support the program’s success. The involvement of a diverse set of program partners through regular meetings, as well as program flexibility and responsiveness, were essential ingredients driving implementation. Projects that engage in self-reflection through document review may support the development of complex programs and build upon existing program guidelines in new and important ways. Findings also offer insights into how to optimize geriatric-surgery co-management and drive a complex initiative forward.

## Supplementary Material

Supplement 1 Supplementary Table 1. ALIGN-CARE areas of assessment and common management recommendations.

## Figures and Tables

**Table 1.** ALIGN-CARE meetings by type, initiation and frequency, attendees, and purpose.

Type	Initiation and frequency	Attendees	Purpose

Interdisciplinary team	Bi-monthly (16 meetings, 16 hours, between 02.26.2020 and 01.2022)	PD, NP, SW, CC, AA	Day-to-day logistics and problem-solving
Advisory	Monthly (28 meetings, 28 hours, between 01.22.2020 and 01.2022)	PD, NP, SW, CC, AA, supervisors	Broader perspectives, strategic input on new protocols, and problem-solving

Surgery team	Monthly (5 meetings, 2.5 hours, between 04.21.2021 and 01.2022)	PD, surgical teams	Surgical team involvement, problem-solving, and buy-in
Research	Weekly (35 meetings, 35 hours, between 05.26.2020 and 01.2022)	PD, research team	Establishing feasible mixed-methods pilot evaluation

PD, program director/geriatrician; NP, nurse practitioner; SW, social worker; CC, care coordinator; AA, administrative assistant; supervisors, department leadership; surgical team, nurse practitioners, clinical assistants, surgeons; research team, lead researchers, program coordinators, assistants.

**Table 2.** Geriatric surgery co-management program components, challenges, elements, and supportive strategies.

Program pathway components	Challenges encountered	Initial and adaptive elements (the “what”)	Supportive implementation strategy (the “how”)

Surgeon screening and referral	Lack of surgeon participation in screenings and referrals Frailty screening quality	Surgeon administered Frail Scale screening *Patient facing flyer	Surgeon needs assessment Surgeon’s Frail Scale training Surgeon facing flyer Informal/formal surgical team meetings
Scheduling	High no-show rate for scheduled visits Lack of patient understanding of visits Short-staffed	Administrator led outreach and scheduling *Scripted appointment scheduling *Reminder calls *Increased appointment slots *Simple scheduling template	Identify as program component No-show tracking, report sharing Commit to ongoing focus in meetings Add dedicated staff

Assessments	Assessment burden Identified need for a SDOH screening	*Refined assessments *Assessments in two-parts	Establish templates to standardize data entry for evaluation purposes Commit to ongoing focus in meetings
Plan	Gap in management plans Need for post-assessment follow-up No community-based social worker to take on management	*“Hotline” for patient communication continuity *Formalized/refined management plan components *Plan communication with surgeon, primary care provider, existing social worker	Ongoing resource review for tailored management plans Commit to ongoing focus in meetings

* Adaptive elements; SDOH, social determinants of health.

## Data Availability

the dataset generated and analyzed is not publicly available because it contains specific information from a clinical program. The codebook, including code examples, will be made available upon reasonable request.
