# Supplementary material for: Developing and implementing a geriatric surgery co-management program for older adults: insights from document analysis
Source: Surg Geriatr Frailty. Author manuscript; Available in PMC 2026 Jan 6. (PMC12766624; doi:10.4081/sigaf.2025.28)
Supplement: Supplement 1 — Supplementary Table 1. ALIGN-CARE areas of assessment and common management recommendations. [file NIHMS2119353-supplement-Supplement_1.pdf]

Surgery in Geriatrics and Frailty

Developing and implementing a geriatric surgery co-management program for older adults: insights from document analysis

Abigail Baim-Lance,[1,2] Fred Ko,[1,2] William Hung,[1,2] Minji Kim,[3] Kavya Sreevalsan,[4] Stephanie Chow[1]

[1]Icahn School of Medicine at Mount Sinai, New York (NY); [2]James J. Peters Department of Veterans Affairs Medical Center, Bronx (NY); [3]University of Minnesota School of Medicine, Minneapolis (MN); [4]Ohio State University Wexner Medical Center, Columbus (OH), USA

doi: 10.4081/sigaf.28

SUPPLEMENTARY MATERIAL

Supplementary Table 1. ALIGN-CARE areas of assessment and common management recommendations.

Area of assessment	Assessment tool	Description	Definition of impairment	Most common geriatric-guided management recommendation in intervention
Overall health and frailty	Frail Scale	Assess patient for pre-frail and frailty	1 or more point	• Geriatrician to provide counseling on patient's frailty risk factors, importance of ALIGN-CARE recommendations<br>• Patient enrolled into ALIGN-CARE program to receive program services (medical, social work, care coordination)
	Clinical Frailty Scale	Assess frailty (done by geriatrician)	Score of 4-9	

Multi-morbidities	Overall comorbid disease burden; specific chronic disease management	Assess degree of comorbidity associated with mortality	Depending on specific chronic disease	• Modify current management plan in preparation for upcoming surgery<br>• Initiate direct communication (written, electronic, or phone) with patient's primary care physician about ALIGN-CARE recommendations re: chronic disease management<br>• Health behavior modifications, such as provide smoking cessation counseling and support if patient currently smokes
Mobility/functional status	Katz ADL	Assess difficulty with the following 6 activities: bathing, dressing, eating, getting in and out of bed/chairs, walking, toileting	Score <4	• Provide fall counseling information<br>• Provide explicit verbal and written instructions on recommendations for balance and gait, resistance training<br>• Referrals: refer to 1) physical therapist (outpatient or home-based); 2) occupational therapist; 3) home attendant services; 4) personal emergency response information; 5) vision specialist if difficulties; 6) hearing specialist if difficulties<br>• Physical examination: check orthostatic blood pressure and decrease or eliminate blood pressure medications if blood pressure is low or low normal<br>• Early referral to inpatient PT for physical frailty/fall risk
	Lawton-Brody IADL	Assess independence in the following 7 activities: using telephone, transportation, shopping, preparing meals, doing housework, taking medicine, managing money	Score <6 (women); <4 (men)	
	Falls history	Assess history of falls and mechanism	A fall within past 3 months	
	Gait speed	Assess mobility over 4 meters; longer time indicates worse performance	Speed >0.8m/s	
	Karnofsky Performance Score	Assess physical functional ability	Score <70 (70 is "cares for self; unable to carry on normal activity or do active work")	
Mind/memory/mood	MoCA	Screen patient for potential cognitive impairment in context of overall education and literacy in English or Spanish language	Score <26 suggestive of possible cognitive impairment	• Provide explicit written instructions for appointments, medications, treatment plan<br>• Medication review: minimize psychoactive and high risk medications<br>• Assess decision-making capacity and elicit health care proxy information and input if patient lacks decision-making capacity<br>• Provide patient/family education on delirium risk, strategies for risk reduction
	Capacity Assessment (4 questions)	Assess for patient's ability for decision-making regarding the surgery (communicating a choice, understanding, appreciation, and rationalization/reasoning)	Any answer "no" would prompt further discussion with patient, healthcare proxy, and primary care provider	

				• Inpatient to identify delirium risk factors for prevention and track occurrence and provide management plan if delirium occurs
	PHQ-2 depression Screen	Assess for depressed mood<br>Positive screen triggers further assessment of symptoms	Positive screen prompts PHQ-9 depression screen	• Referral: refer to 1) counseling or psychotherapy; 2) social work; 3) spiritual counseling or chaplaincy services; 4) psychiatry if severe symptoms or already on medications that are inadequate; 5) palliative care<br>• Initiate pharmacologic therapy if appropriate in conjunction with primary care provider<br>• Provide linkage to community resources (such as support groups and local/national programs)
Medication polypharmacy	Anti-Cholinergic Burden Scale	Assess for anticholinergic burden	Any point would result in discussion with patient and written recommendation to PCP	• Ask patient to bring in prescribed, over the counter medications, supplements<br>• Contact prescriber (usually primary care provider) for adjustments to avoid potentially inappropriate medications and reduce regimen complexity<br>• Consult pharmacist who fills scripts to synchronize medication refills whenever possible<br>• Consult pharmacist who fills scripts to blister pack if deemed helpful<br>• Recommend pillbox and/or medication calendar, or blister packing via pharmacy<br>• Provide education and review of potential targets for deprescribing
	Beers List	Assess for potentially inappropriate medications	Discussion with patient and written recommendation to PCP	
	Lexicomp drug interaction calculator	Assess for drug interactions	Drug interaction rating of "D" (recommend avoid) or "X" (recommend stopping)	
Nutrition	Mini Nutritional Assessment	Assess for preoperative adequacy of nutrition	Score <14 triggers referral to nutritionist and patient hand-out for dietary recommendations	• Provide nutrition hand-out<br>• Referrals: refer to 1) nutritionist/clinical dietician; 2) dentist if poor dentition or chewing issues; 3) speech and swallow if difficulties swallowing<br>• Provide written documentation to inpatient teams
Goals of care discussion	Health Care Proxy discussion and form completion	Assess for preemptive advance care planning just prior to surgery	Refusal to complete form triggers outreach to primary care provider for follow-up/rapport	• Align treatment plan with stated goals<br>• Provide written documentation to primary care physician, surgeon, and inpatient teams

	Health outcome prioritization	Assess for preemptive advance care planning values just prior to surgery	Refusal to complete triggers further exploration to “What matters most” to patient	• Discussions with surgeon, PCP, patient/healthcare proxy as appropriate
Surgical risk	NSQIP Risk Calculator	Assess for predicted adverse outcomes post-surgery	N/A	• Provide written documentation to inpatient teams regarding predicted surgical outcomes from NSQIP data<br>• Provide peri- and post-operative anticipated cardiac morbidity and mortality precautions
	Goldman’s RCRI	Assess for predicted cardiac mortality and morbidity outcomes post-surgery	Score 2+ is “elevated cardiac risk”	
Maintaining care/social determinants of health	CMS survey	Assess SDOH in 13 domains: Food, housing, income, literacy, social supports, safety, legal, transportation, healthcare, medication, mental health, substance use/disability	Determine “need identified” by social work triggers further recommendations to primary care provider/primary social worker.	• Social worker to provide explicit verbal and written recommendations to patient and primary care clinician<br>• Example Referrals: refer to: 1) Medicaid REAP office for Medicaid services including home attendant care; 2) meals program for home delivery food service; 3) state-wide memory care program<br>• Preparation of care partners for surgery and after care

ADL, activities of daily living; PT, physical therapy; IADL, instrumental activities of daily living; MoCA, Montreal Cognitive Assessment; PHQ-2, Patient Health Questionnaire-2; PCP, primary care provider; NSQIP, National Surgery Quality Improvement Program; RCRI, Revised Cardiac Risk Index; CMS, Center for Medicare and Medicaid Services.
